# The Effect of *Lactobacillus acidophilus* YT1 (MENOLACTO) on Improving Menopausal Symptoms: A Randomized, Double-Blinded, Placebo-Controlled Clinical Trial

**DOI:** 10.3390/jcm9072173

**Published:** 2020-07-09

**Authors:** Eun Yeong Lim, So-Young Lee, Hee Soon Shin, Jaekwang Lee, Young-Do Nam, Dong Ock Lee, Ji Young Lee, Sung Hum Yeon, Rak Ho Son, Chae Lee Park, Yun Haeng Heo, Yun Tai Kim

**Affiliations:** 1 Division of Food Functionality Research, Korea Food Research Institute, Wanju 55365, Korea; 50005@kfri.re.kr (E.Y.L.); sylee09@kfri.re.kr (S.-Y.L.); hsshin@kfri.re.kr (H.S.S.); jklee@kfri.re.kr (J.L.); youngdo98@kfri.re.kr (Y.-D.N.); 2 Department of Food Biotechnology, Korea University of Science & Technology, Daejeon 34113, Korea; 3 Center for Gynecologic Cancer, National Cancer Center Korea, Goyang-si 10408, Korea; dolee@ncc.re.kr; 4 Department of Obstetrics and Gynecology, Konkuk University Hospital, Konkuk University School of Medicine, Seoul 05030, Korea; jylee@kuh.ac.kr; 5 R&D Center, Huons Co., Ltd., Ansan 15588, Korea; yon3547@huons.com (S.H.Y.); sonnaco@huons.com (R.H.S.); cherry890709@huons.com (C.L.P.); 6 Clinical Operation Team, Huons Co., Ltd., Seongnam-si 13486, Korea; gj0221@huons.com

**Keywords:** MENOLACTO, *Lactobacillus acidophilus* YT1, menopausal symptoms, clinical trial, Kupperman index

## Abstract

This study evaluated the efficacy of *Lactobacillus acidophilus* YT1 (MENOLACTO) for alleviating menopausal symptoms. This study was a multi-center, randomized, double-blinded, placebo-controlled clinical trial involving female subjects (ages: 40–60 years) with menopausal symptoms and a Kupperman index (KMI) score ≥ 20. Subjects were administered 1 × 10^8^ CFU/day MENOLACTO or placebo, with the primary endpoint being total KMI score, and the effect of secondary endpoints on alleviating menopausal symptoms according to individual categories of the modified KMI, as well as a quality of life questionnaire (MENQOL questionnaire). After 12 weeks, total KMI scores decreased significantly, demonstrating improved menopausal symptoms relative to placebo along with improved modified KMI scores. Additionally, quality of life, according to the MENQOL questionnaire, significantly improved in all four symptoms—physical, psychosocial, vasomotor, and sexual symptoms. Moreover, we observed no significant difference between the two groups or significant changes in blood follicle-stimulating hormone and estradiol levels or endometrial thickness. These results demonstrated that MENOLACTO alleviated menopausal symptoms without notable side effects and improved quality of life, suggesting its efficacy as an alternative supplement to alleviate menopausal symptoms in women ineligible for hormonal therapy.

## 1. Introduction

Menopause is a natural physiological status in women caused by loss of ovarian follicular-reproduction function through aging and diagnosed following the absence of menstrual periods for 12 consecutive months. Menopause occurs in most women between the ages of 45 and 55, although timing and symptoms can vary depending on the individual. Menopausal symptoms are increasingly recognized as having significant health implications through decreases in estrogen levels and lower estrogen exposure [1,2]. Major symptoms include vasomotor issues (hot flushes and/or night sweats), sleep disruption, vaginal symptoms (dryness and/or infections), depression and anxiety, cognitive changes (declines in memory and concentration), and sexual dysfunction [3,4].

According to the World Health Organization (WHO), the average life expectancy of women is >80 years in at least 35 countries. Given the increases in life expectancy, many women typically spend more than one-third of their entire life in a menopausal state [5]. Moreover, the population of menopausal women is projected to increase to 1.2 billion by 2030 due to projected annual increases of 47 million new menopausal women [6]. However, because menopause and its symptoms represent a known physiological phenomenon, there is a lack of awareness regarding symptom prevention and treatment.

Currently, treatments for menopausal symptoms include non-pharmaceutical therapy (e.g., lifestyle changes through exercise and diet), phytoestrogen therapy [7], and hormone and non-hormone therapies (serotonin-reabsorbing inhibitors [8] and gabapentin [9], respectively). Among these, hormone-replacement therapy (HRT) is the most effective treatment for menopausal symptoms and used to alleviate physical symptoms associated with estrogen deficiency and prevent the development of vasomotor symptoms, sleep disruption [10,11], and vaginal dryness [12,13]. Additionally, most phytoestrogens obtained from natural extracts have chemical structures and activities similar to estrogen, making them capable of addressing vasomotor symptoms [14,15] and potentially improving psychiatric and sexual symptoms [16]. Moreover, HRT and phytoestrogen therapies reportedly improve neuroprotective effects [17,18], rheumatoid arthritis [19], and osteoporosis [20] in menopausal women. However, previous studies have also reported that long-term HRT can cause side effects, including breast cancers, cerebrovascular disease, and thromboembolism [21,22,23]. Furthermore, there is a lack of solid evidence supporting the routine use of phytoestrogen to improve postmenopausal quality of life [24], and the activities of herbal preparations have not been sufficiently investigated, with limited data available regarding their safety [25].

Therefore, new approaches to the treatment of menopause are needed. Interest has recently increased in the microbiome [26,27,28], with studies focused on identifying a safe, potent, and non-toxic drug and functional food source from the intestinal microbiome. The intestinal microbiome is not only related to improved digestive function but also various menopause-related symptoms, including sleep disturbance [29], depression/psychiatric health [30], and bone health [31]. According to a previous report, oral and gut microbiota affected by estrogen correlate with menopausal symptoms (obesity, osteoporosis, and cancer) [32], and there are reports that the abundance and diversity of intestinal microorganisms affect estrogen metabolites in women after menopause [33].

We recently identified changes in intestinal microbes between a control group and a menopause animal model, with notable reductions observed in *Lactobacillus acidophilus* YT1 (MENOLACTO^®^) [34,35]. However, no data have been published on the ability of MENOLACTO^®^ to improve symptoms in menopausal women. To evaluate the efficacy and safety of MENOLACTO^®^ in this context, we conducted a double-blinded, randomized, placebo-controlled clinical trial over the course of 12 weeks.

## 2. Experimental Section

### 2.1. Study Design

This study was a 12-week, multi-center, randomized, double-blinded, placebo-controlled human study that evaluated the effect of live *L. acidophilus* YT1 (1 × 10^8^ colony-forming units (CFU)/day) on improving menopausal symptoms according to the Kupperman index (KMI). The study was conducted with the approval of an Institutional Review Board at the National Cancer Center and Konkuk University Hospital and commenced on 12 June 2017 and ended on 7 March 2018.

Eligible subjects were allocated to each group via block randomization in the ratio of 1:1. The random sequences were generated using SAS software. Subjects were randomly assigned using SAS version 9.4 (SAS Institute, Cary, North Carolina, USA) software.

Subjects underwent four visits, including the screening visit (visit 1), and participated in the study at 6-week intervals from baseline (visit 2) for a total duration of 12 weeks. MENOLACTO^®^ was manufactured by modifying the previous method [35]. The lactic acid bacteria strain was *L. acidophilus* YT1 (KCCM11808P), and it was deposited at the Korea Culture Center of Microorganisms (KCCM) under number KCCM11808P. The MENOLACTO^®^ contained the *L. acidophilus* YT1 (1 × 10^8^ CFU, 15%), isomalt (64%), xylitol (14%), and yogurt powder (7%) in stick, and control product (placebo) contained isomalt (79%), xylitol (14%), and yogurt powder (7%). Both the MENOLACTO^®^ and placebo had a similar appearance, texture, and taste. Both products were specifically prepared for the study and provided by the Bio-farm Co., Ltd. (Gyeonggi-do, Korea). During the study period, subjects received the experimental product (MENOLACTO^®^) or a control product (placebo) with or without water.

**Figure 1:**
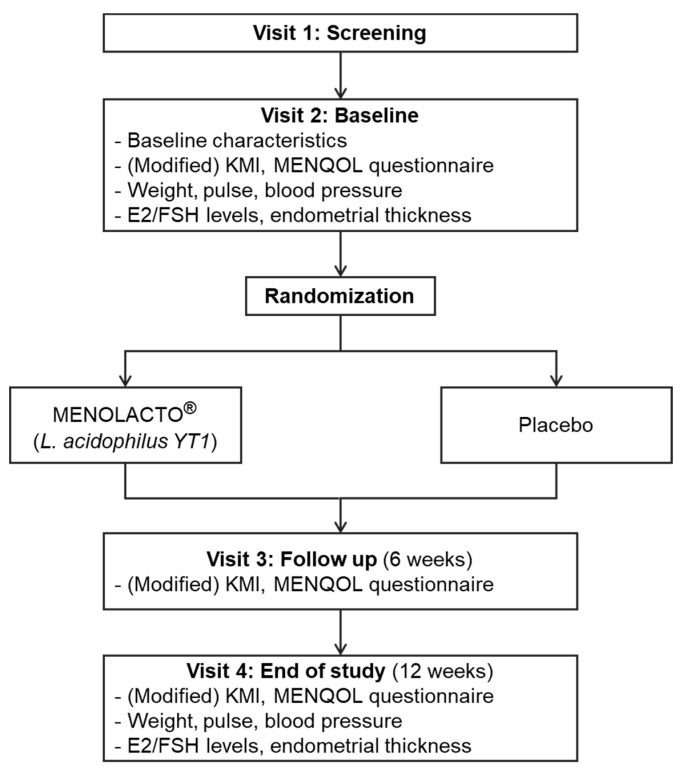
Study schema.

Subjects received an explanation of the study goals and provided written informed consent on visit 1 prior to study participation. Subjects underwent a primary compatibility evaluation based on demographic characteristics, medical history, medication history, physical examination, vital signs, body measurements, clinical laboratory tests, mammography/papanicolaou test, and modified KMI (11 items on the KMI assessing vaginal dryness/decreased discharge) and received a date for their follow-up visit (within 14 days after visit 1). Subjects were evaluated for changes in medical or medication history and underwent a physical examination. The results of the first and second evaluations were pooled to determine compatibility with inclusion/exclusion criteria, after which subjects underwent randomization. After randomization, subjects underwent blood and urine tests to allow evaluation of efficacy and safety, as well as a quality of life evaluation. Subjects received explanations concerning the measurement process for determining the endometrial thickness and the prescription and administration of MENOLACTO^®^. The MENOLACTO^®^ and placebo subjects took one MENOLACTO^®^ stick (1 × 10^8^ CFU/day) and placebo per dosing one time a day for 12 weeks. In this study, intake of various foods and drugs that could interfere with the results, such as hormone therapy, hormone analog (e.g., plant extract), probiotics, anti-depressant agents, anti-convulsant agents, anti-anxiety drugs, and osteoporosis drugs, was excluded in the instructions regarding diet. Visits 3 and 4 occurred at 6-week intervals for evaluations of adverse events, effects of co-administered medications, physical examination, vital signs, body measurements, blood and urine tests, quality of life evaluation, and KMI scoring (Figure 1).

The primary endpoint was a change in total KMI score before and after the 12-week administration of the MENOLACTO^®^. Secondary endpoints were modified KMI scores of individual items (11 items from the KMI and vaginal dryness) and quality of life assessment [36,37]. For safety evaluation, we analyzed adverse events, clinical laboratory findings (hematological test/blood chemistry and urine tests), vital signs, physical measurements, estradiol (E2) and follicle-stimulating hormone (FSH) levels, and endometrial thickness.

### 2.2. Subjects

The research protocols were approved by the Institutional Review Board of Konkuk University hospital (IRB No. KUH1040060; date: 11 May 2017; Seoul, Korea) and National Cancer Center Korea (IRB No. NCC2017-0114; date: 11 May 2017; Goyang, Korea). All participants were provided with a full explanation of the study procedures and provided written informed consent before entering the clinical trial. All methods were conducted according to relevant guidelines, and the protocol was approved by the Institutional Review Board of Konkuk University hospital and National Cancer Center Korea. This study was registered in the WHO International Clinical Trials Registry Platform (KCT0003211; date: 21 September 2018).

**Figure 2:**
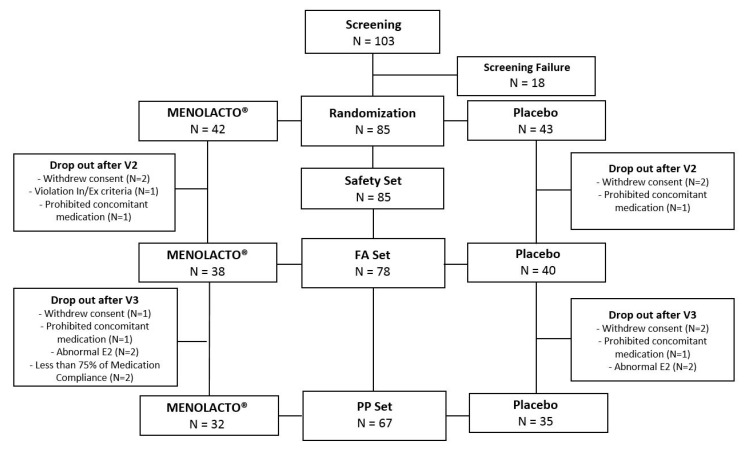
The flow chart for the subject’s enrollment, randomization, and retention.

Menopausal women experiencing peri-menopausal symptoms (*n* = 103) were recruited as study subjects and screened, of which 18 were excluded, and 85 underwent randomization (42 in the experimental group and 43 in the control group). Six subjects were excluded for withdrawal of consent (3 from the experimental group and 3 from the control group), two for taking medications contraindicated for co-administration (1 from each group), and one from the control group for failure to follow-up. A total of 67 subjects completed this study (32 from the experimental group and 35 from the control group). The flow of subjects is shown in Figure 2.

### 2.3. Inclusion and Exclusion Criteria

Subjects who met inclusion criteria were included: (1) women aged between 40 and 60 years who had been amenorrheic for ≥ 1 year or had undergone hysterectomy with follicle-stimulating hormone (FSH) levels of ≥ 30 mIU/mL, (2) Kupperman index (KMI) score of ≥ 20, (3) volunteers in this trial with a written informed consent form.

Subjects with the following criteria were excluded: (1) women with a body mass index > 30 Kg/m^2^, (2) use of hormonal therapy or a hormone analog (e.g., plant extract) within 3 months, (3) history of endometrial hyperplasia, uterine or endometrial cancer, breast disease, or cancers associated with sex hormones, (4) history of severe migraines within 1 year, (5) thromboembolism, cerebrovascular disease, myocardial infarction, unstable angina, or previous coronary angioplasty, (6) history of severe psychological diseases, such as depression or anxiety, or use of psychiatric drugs, such as anti-depressants, (7) and a history of abnormal uterine bleeding 1 year after menopause.

### 2.4. Outcome Measurements

A change in the total KMI score between baseline and that after 12 weeks of administration was the primary endpoint, and the secondary endpoints were the 12 items of the modified KMI, including vaginal dryness [36,37]. The KMI was created to increase the efficiency of treatment for this syndrome by characterizing the severity and characteristics of menopausal disorder through the sum of indices of major menopausal symptoms. KMI scores at visits 1, 3, and 4 are widely used in Japan and elsewhere to evaluate peri-menopausal syndrome. The KMI includes 11 symptoms (hot flashes, paresthesia, insomnia, nervousness, depression, dizziness, fatigue, musculoskeletal pain, headache, palpitation, and formication). Each of the 11 items is evaluated on a 4-point scale (0–3) (0 = not at all, 1 = weak, 2 = moderate, 3 = severe symptoms) depending on symptom severity, with each item weighted individually corresponding to importance symptom. For hot flashes, the score was multiplied by 4; for paresthesia, insomnia, and nervousness, the score was multiplied by 2; for other symptoms, including depression, dizziness, fatigue, musculoskeletal pain, headache, palpitation, and formication, there was a weighting of 1. In this study, the KMI evaluation was performed at visit 1 (screening; week 2). A KMI score (for the 11 items) of ≥ 20 was included for analysis.

Secondary endpoints were assessed by modified KMI and MENQOL questionnaire. The modified KMI reportedly shows a high correlation with menopausal symptoms, thereby making it valuable for their evaluation [38]. The MENQOL questionnaire was used to evaluate the quality of life, including 29 questions in terms of vasomotor (3 items), psychological (7 items), physiological (16 items), and sexual symptoms (3 items) [39]. MENQOL reflects the quality of life during the peri-menopausal period. MENQOL questionnaire has the seven-point Likert scale, ranging from 0 (not at all bothered) to 6 (extremely bothered) points. Once each item was reported as a 0–6 score, the average value was calculated for each domain.

### 2.5. Sample Size

This was the first study to evaluate the efficacy of *L. acidophilus* YT1 in menopausal women. Then, we selected the previous clinical study, which showed a significant efficacy on the KMI used in our study [38]. The difference in mean change was −4.4 in sample size formula. We estimated that a sample of 34 postmenopausal women for each group would be required to detect a difference with a power of 80% and a two-sided α level of 5%. Considering a 15% sample loss, the necessary sample size was determined to be 40 participants for each group.

### 2.6. Statistical Analysis

We used the per-protocol analysis set (PPS) as the main analysis set for efficacy evaluation, and according to the intention-to-treat principle that includes all randomized subjects in the analysis, the full analysis set (FAS) comprised the study subjects receiving at least one dose of the investigational product, undergoing at least one efficacy evaluation, and not meeting the major exclusion criteria. The PPS comprised study subjects included in the FAS set who completed this study and did not have a serious violation (e.g., a violation of inclusion/exclusion criteria) that might affect the study results. The PPS included 67 subjects (32 experimental and 35 control subjects).

For the statistical analysis of efficacy, we used a paired *t*-test to analyze the primary endpoint. Statistically significant differences between the two groups according to the degree of change were evaluated using a two-sample *t*-test, the Wilcoxon rank-sum test, and analysis of covariance (ANCOVA).

For the evaluation of the modified KMI scores (presented as the mean ± standard deviation (SD); 11 KMI items and vaginal dryness/decreased discharge) according to individual items and changes in the MENQOL questionnaire before and after administration, we used a paired *t*-test. Statistically significant differences between the two groups according to the degree of change were analyzed using a two-sample *t*-test, the Wilcoxon rank-sum test, and ANCOVA.

Safety evaluation was conducted using the safety set, which comprised subjects administered at least one dose of MENOLACTO^®^ after randomization (42 experimental and 43 control subjects). The safety set was evaluated for adverse events, vital signs, and E2 and FSH levels. The incidence rate of adverse events was calculated for each group, and a chi-squared test or Fisher’s exact test was used for comparative analysis. A two-sample *t*-test was used for comparisons of vital signs, E2 and FSH levels, and endometrial thickness between the two groups.

## 3. Results

### 3.1. Characteristics of Study Patients

The experimental group and control group had no significant difference in demographic information between marital status, physical activity, stress awareness, weight, pulse, and systolic and diastolic blood pressure. Demographic information is shown in Table 1.

### 3.2. Primary Efficacy Endpoint

#### Total KMI Score

Analysis of changes in total KMI score showed that after 6 weeks of administration of MENOLACTO^®^, scores decreased relative to baseline by 17.47 ± 11.00 in the MENOLACTO^®^ group (*p* < 0.0001) and by 8.29 ± 8.94 in the control group (*p* < 0.0001). Additionally, the total KMI score at 12 weeks decreased relative to baseline by 21.50 ± 12.75 in the MENOLACTO^®^ group (*p* < 0.0001) and by 12.14 ± 9.83 in the control group (*p* < 0.0001). These results indicated a significant difference between the two groups at both 6 and 12 weeks (Table 2).

### 3.3. Secondary Efficacy Endpoints

#### 3.3.1. Change in Modified KMI Score by Category

Table 3 shows the change in scores for the 12 categories of modified KMI (including vaginal dryness). We found statistically significant differences at 12 weeks between the experimental and control groups in evaluations of hot flashes, paresthesia, nervousness, depression, fatigue, musculoskeletal pain, headache, heart palpitation, formication, and vaginal dryness/decreased discharge (Table 3).

#### 3.3.2. Evaluation of the Quality of Life

To evaluate the quality of life, subjects completed the MENQOL questionnaire, with the answers used to measure subjective improvement. The questionnaire comprises 29 questions, assessing physical, psychosocial, vasomotor, and sexual symptoms. For this evaluation, a decrease in the MENQOL score represented an improvement in the quality of life. The analysis of changes in MENQOL scores revealed that all four symptom categories showed statistically significant differences between the two groups at 6 and 12 weeks of MENOLACTO^®^ administration (Table 4.).

### 3.4. Safety

#### 3.4.1. Adverse Events

Safety was evaluated in the safety set, with 12 adverse events from eight subjects in the experimental group and 10 adverse events from eight subjects in the control group reported, although the difference between the two groups was not statistically significant. One serious adverse event occurred in the control group, and there was no discontinuation due to adverse events (Table 5).

Adverse events occurring after administration of MENOLACTO^®^ were coded according to System Organ Class (MedDRA; https://www.meddra.org/). Major adverse events in the experimental group included neurological disease, reproductive or breast dysfunction, respiratory dysfunction, and thoracic or mediastinal disease, whereas those in the control group included respiratory dysfunction and thoracic or mediastinal disease. A serious adverse event occurred in one subject in the control group with a history of hospitalization due to pneumonia prior to this study. The subject showed recurrence of pneumonia symptoms during the study and for which the subject was hospitalized; however, the subject recovered with standard treatment and was discharged. The investigators deemed that there was no evident causal relationship between the serious adverse event and MENOLACTO^®^ administration. The subject was subsequently excluded from the study due to failure to follow-up after visit 3.

Evaluation of the severity of symptoms associated with the adverse events revealed that the experimental group showed 11 cases of mild adverse events and one case of a moderate event, whereas the control group showed nine cases of mild events and one moderate event. Five cases in the experimental group were determined as having no association with MENOLACTO^®^, four were deemed evidently unassociated with MENOLACTO^®^, and three were deemed to have an unknown association with MENOLACTO^®^. In the control group, three cases were determined as having no association with MENOLACTO^®^, and seven were deemed evidently unassociated with MENOLACTO^®^. There were no statistically significant differences between the two groups (*p* = 0.1812).

#### 3.4.2. Changes in E2 and FSH Levels

The experimental group showed a decrease in E2 level by 11.11 ± 52.92 pg/mL after 12 weeks of MENOLACTO^®^ administration, whereas the control group showed a decrease of 9.16 ± 49.10 pg/mL, although the difference between the two groups was not significant (*p* = 0.8678). Additionally, the experimental group showed an increase in FSH level of 3.80 ± 25.02 mIU/mL after 12 weeks of MENOLACTO^®^ administration, whereas the control group showed an increase of 5.96 ± 33.74 mIU/mL, with this difference also not statistically significant (*p* = 0.7526) (Table 6).

#### 3.4.3. Changes in Endometrial Thickness

Endometrial thickness decreased in the experimental group by 0.31 ± 2.01 mm after 12 weeks of MENOLACTO^®^ administration, whereas the control group showed an increase of 0.24 ± 1.48 mm, although the difference between the two groups was not statistically significant (*p* = 0.2091) (Table 7).

## 4. Discussion

This WAs the first clinical trial to simultaneously investigate the efficacy and safety of a 12-week treatment with *L. acidophilus* to improve symptoms in menopausal women. In this multi-center, randomized, double-blinded, placebo-controlled clinical trial, MENOLACTO^®^ significantly reduced menopausal symptoms according to instruments commonly used to measure outcomes (total KMI score and MENQOL). The KMI score revealed a ~66% reduction after MENOLACTO^®^ treatment relative to baseline levels, whereas a 37% reduction was observed in the placebo group. Regarding specific symptoms, MENOLACTO^®^ treatment alleviated symptoms, such as hot flashes, paresthesia, nervousness, melancholia, fatigue, arthralgia, headache, heart palpitation, formication, and vaginal dryness, according to KMI scores and physical, psychosocial, vasomotor, and sexual symptoms of MENQOL responses relative to the placebo group.

HRT relieves menopausal symptoms by restoring sex hormone levels [40]; however, long-term use of hormone therapy is associated with an increased risk of coronary heart disease, thromboembolic events, stroke, and breast malignancy [41]. Phytoestrogens, proposed as an HRT alternative, have been evaluated for their efficacy in treating menopausal symptoms [42,43]; however, study findings using menopausal women are insufficient and show conflicting results [44,45]. Recent studies show that menopausal symptoms are alleviated independent of E2 concentration in vivo by regulating gut microbiota [46,47]. Furthermore, probiotics might influence both the enteric nervous system and the central nervous system in addition to their effects on the mucosal immune system through modification of the gut microbiome [48]. Therefore, in the present study, we assessed the ability of the probiotic *L. acidophilus* to relieve menopausal symptoms.

Gut microbiota significantly affects metabolism, immunity, and protection against pathogens [49]. The relationship between gut microorganisms and menopause has been previously studied [32], with findings revealing that gut microflora changes after menopause in both animal models and clinical trials [50,51]. Intestinal microbial changes might represent a mechanism through which menopausal symptoms can be alleviated, regardless of estrogen level. A previous study has reported that sex hormone deficiency increases gut permeability and levels of osteoclastogenic cytokine, resulting in bone loss; however, this has not been observed in germ-free mice [52]. By contrast, sex hormone treatment alters gut microbiota [53,54], with several studies reporting that menopausal symptoms are alleviated by regulating gut microbiota [47,55]. Probiotics are well-known regulators of gut microbiota and improve bone mineral density in menopause animal models by regulating inflammation [52,56]. Additionally, probiotics reduce depressive behavior by regulating inflammation and activity by the hypothalamus–pituitary–adrenal axis [57]. In the present study, menopausal symptoms were alleviated, regardless of E2 and FSH levels, suggesting that MENOLACTO^®^ relieved climacteric symptoms.

There are a limited number of clinical trials of probiotics assessing their effect on menopausal women. Probiotics are currently being studied in relation to vaginal and bone health. A previous study has shown that in postmenopausal women, Nugent scores decrease after a 14-day treatment with *Lactobacillus reuteri* RC-14 and *Lactobacillus rhamnosus* GR-1 along with sodium alginate [58]. Additionally, vaginal maturation indices reportedly change following administration of *L. acidophilus* and estriol tablets to postmenopausal women with vaginal atrophy symptoms [59]. Moreover, a mixture of *Lactobacillus* strains improves lumbar spine-bone loss in postmenopausal women [60]. However, there are no studies directly addressing whether probiotics relieve menopausal symptoms, although clinical trials have been conducted in relation to symptoms of insomnia, depression, fatigue, and headache [61,62,63,64].

In this study, participants who received MENOLACTO^®^ treatment for 12 weeks showed a significant reduction in scores related to all KMI categories relative to baseline scores. Compared with a previous study reporting similar baseline KMI scores to those reported here, hormone therapy, along with individualized pharmaceutic care for 12 months, resulted in similar reductions in KMI scores to those reported following MENOLACTO^®^ treatment for 12 weeks [65]. This suggested that the reduced KMI levels associated with MENOLACTO^®^ treatment are clinically relevant.

Our data showed that MENOLACTO^®^ treatment did not alter endometrial thickness, and we observed no elevation in circulating levels of E2. These results supported the therapeutic safety of MENOLACTO^®^, although further studies are required to assess its long-term effects on the endometrium. This study had some limitations. First, KMI scores and the MENQOL questionnaire are widely used to diagnose menopause; however, these methods rely on accurate self-reporting. Second, the follow-up was for a relatively short period, and the mechanism of action remains unclear. We suggested that intestinal microbial regulation might be involved in the observed results; however, the intestinal microbial analysis was not conducted. Further studies are needed to confirm the effectiveness of MENOLACTO^®^ therapy in menopausal women, as well as the associated mechanism.

## 5. Conclusions

In summary, we reported an improvement in menopausal symptoms following MENOLACTO^®^ treatment according to the results of a multi-center, randomized, 12-week, double-blinded, placebo-controlled clinical trial. The findings demonstrated that MENOLACTO^®^ treatment was effective at alleviating menopausal symptoms and was safe, with no adverse effects on E2 levels. Additionally, this was the first study demonstrating improvements in total KMI scores, as well as scores in 10 KMI categories, relative to a placebo group. Therefore, these findings suggested the efficacy of MENOLACTO^®^ as a dietary supplement for women experiencing menopausal symptoms, such as hot flashes, paresthesia, nervousness, melancholia, fatigue, arthralgia, headache, heart palpitation, formication, and vaginal dryness.

## Figures and Tables

**Table 1:** Characteristics of the study population.

Domain		*n*	MENOLACTO^®^	*n*	Placebo	*p*
Age	Baseline	42	54.48 ± 3.79	43	52.40 ± 3.89	0.0195 *
E2 (pg/mL)	Baseline	42	28.10 ± 47.78	43	25.82 ± 43.59	0.8189 *
FSH (mIU/mL)	Baseline	42	75.29 ± 33.91	43	74.02 ± 30.38	0.8561 *
Endometrial thickness (mm)	Baseline	37	2.78 ± 1.68	39	2.56 ± 1.19	0.4974 *
Total KMI score	Baseline	38	32.92 ± 9.15	40	32.73 ± 9.53	0.9265 *
Marital status	Unmarried	42		43	1	1.0000 ^†^
	Married		42		41	
	Divorced				1	
Physical activity	No	42	14	43	14	0.9205 **
	Low		13		15	
	High		15		14	
Stress awareness (%)	None	42	2	42	2	0.9032 ^†^
	Moderate		20		24	
	High		16		14	
	Very high		4		3	
Weight (kg)	Baseline	42	56.26 ± 6.94	43	57.66 ± 9.50	0.4382 *
Pulse (beats/min)	Baseline	42	72.69 ± 11.76	43	72.05 ± 9.46	0.7814 *
Systolic blood pressure (mmHg)	Baseline	42	119.14 ± 17.14	43	117.93 ± 15.47	0.7328 *
Diastolic blood pressure (mmHg)	Baseline	42	71.74 ± 10.34	43	71.53 ± 9.83	0.9262 *

Values are presented as the mean ± SD. * Comparison between groups; two-sample *t*-test. ** Comparison between groups; Chi-square test. † Comparison between groups; Fisher’s exact test.

**Table 2:** Change in total KMI score relative to baseline.

Domain		MENOLACTO^®^ (*n* = 32)	Placebo (*n* = 35)	*p* *	*p* **	*p* ^†^
Total KMI score	Baseline	32.75 ± 9.23	33.20 ± 9.87			
	Week 6	15.28 ± 7.64	24.91 ± 13.75	0.0004	0.0002	0.0002
	Week 12	11.25 ± 8.25	21.06 ± 11.31	0.0012	0.0014	0.0005

Values are presented as the mean ± SD. * Comparison between groups (changes from baseline); two-sample *t*-test. ** Comparison between groups (changes from baseline); Wilcoxon rank-sum test. † Comparison between groups (changes from baseline); ANCOVA adjusted for baseline and age.

**Table 3:** Changes in modified KMI scores from baseline according to category.

Domain	MENOLACTO^®^ (*n* = 32)	Placebo (*n* = 35)	*p* *	*p* **	*p* ^†^
Hot flashes					
Baseline	8.13 ± 3.44	9.03 ± 2.63			
Week 6	4.38 ± 2.56	5.83 ± 3.80	0.5376	0.2576	0.1004
Week 12	2.50 ± 2.44	5.14 ± 3.15	0.0526	0.0512	0.0018
Paresthesia					
Baseline	3.75 ± 1.88	3.37 ± 1.66			
Week 6	1.25 ± 1.50	2.63 ± 2.04	0.0002	0.0002	<0.0001
Week 12	1.38 ± 1.64	2.17 ± 1.90	0.0333	0.0356	0.0320
Insomnia					
Baseline	3.94 ± 1.79	4.29 ± 1.82			
Week 6	2.19 ± 1.93	3.37 ± 2.10	0.0515	0.0418	0.0238
Week 12	1.94 ± 1.93	2.91 ± 1.90	0.1841	0.1300	0.1614
Nervousness					
Baseline	4.00 ± 1.61	3.71 ± 2.12			
Week 6	1.81 ± 1.64	3.03 ± 2.08	0.0026	0.0025	0.0014
Week 12	1.13 ± 1.24	2.34 ± 1.91	0.0041	0.0038	0.0026
Melancholia					
Baseline	2.00 ± 0.84	1.97 ± 0.86			
Week 6	0.91 ± 0.69	1.60 ± 0.95	0.0024	0.0053	0.0004
Week 12	0.63 ± 0.66	1.26 ± 0.95	0.0107	0.0131	0.0056
Vertigo					
Baseline	1.38 ± 0.87	1.54 ± 0.82			
Week 6	0.63 ± 0.66	1.20 ± 0.99	0.0568	0.0591	0.0157
Week 12	0.56 ± 0.72	1.03 ± 0.86	0.2806	0.2126	0.0689
Fatigue					
Baseline	2.31 ± 0.74	2.54 ± 0.70			
Week 6	1.13 ± 0.87	1.97 ± 1.01	0.0105	0.0151	0.0016
Week 12	1.00 ± 0.72	1.83 ± 0.92	0.0059	0.0104	0.0028
Arthralgia, Myalgia					
Baseline	2.13 ± 0.71	1.91 ± 0.89			
Week 6	1.16 ± 0.77	1.49 ± 0.92	0.0138	0.0112	0.0115
Week 12	0.91 ± 0.73	1.40 ± 1.03	0.0020	0.0039	0.0033
Headache					
Baseline	1.72 ± 0.81	1.63 ± 1.03			
Week 6	0.75 ± 0.84	1.31 ± 1.02	0.0064	0.0089	0.0080
Week 12	0.47 ± 0.67	1.09 ± 0.85	0.0034	0.0047	0.0028
Heart palpitation					
Baseline	1.81 ± 0.82	1.80 ± 0.93			
Week 6	0.63 ± 0.66	1.34 ± 1.06	0.0009	0.0007	0.0005
Week 12	0.38 ± 0.55	0.94 ± 0.97	0.0180	0.0107	0.0132
Formication					
Baseline	1.59 ± 0.80	1.40 ± 0.98			
Week 6	0.47 ± 0.67	1.14 ± 0.91	<0.0001	<0.0001	<0.0001
Week 12	0.38 ± 0.55	0.94 ± 0.84	0.0019	0.0015	0.0014
Dryness of vagina					
Baseline	2.28 ± 0.89	2.17 ± 1.01			
Week 6	1.25 ± 0.80	1.97 ± 0.95	0.0029	0.0045	0.0003
Week 12	0.94 ± 0.91	1.83 ± 0.92	0.0006	0.0007	0.0003

Values are presented as the mean ± SD. * Comparison between groups (changes from baseline); two-sample *t*-test. ** Comparison between groups (changes from baseline); Wilcoxon rank-sum test. † Comparison between groups (changes from baseline); ANCOVA adjusted for baseline and age.

**Table 4:** Changes in MENQOL scores from baseline according to symptoms.

Domain	MENOLACTO^®^ (*n* = 32)	Placebo (*n* = 35)	*p* *	*p* **	*p* ^†^
Physical					
Baseline	3.36 ± 1.41	3.67 ± 1.33			
Week 6	1.93 ± 1.11	3.36 ± 1.52	0.0018	0.0070	0.0001
Week 12	1.62 ± 1.14	2.72 ± 1.48	0.0481	0.1488	0.0074
Psychosocial					
Baseline	3.39 ± 1.61	3.44 ± 1.37			
Week 6	2.14 ± 1.25	3.34 ± 1.52	0.0037	0.0106	0.0002
Week 12	1.59 ± 1.26	2.72 ± 1.35	0.0218	0.0974	0.0027
Vasomotor					
Baseline	3.84 ± 1.95	3.95 ± 1.81			
Week 6	2.13 ± 1.35	3.40 ± 1.95	0.0072	0.0126	0.0015
Week 12	1.37 ± 1.29	2.72 ± 1.85	0.0129	0.0406	0.0041
Sexual					
Baseline	4.53 ± 1.63	4.22 ± 1.85			
Week 6	2.88 ± 1.76	4.33 ± 1.81	0.0001	0.0003	0.0002
Week 12	2.59 ± 2.00	3.87 ± 2.05	0.0042	0.0057	0.0079

Values are presented as the mean ± SD. * Comparison between groups (changes from baseline); two-sample *t*-test. ** Comparison between groups (changes from baseline); Wilcoxon rank-sum test. † Comparison between groups (changes from baseline); ANCOVA adjusted for baseline and age.

**Table 5:** Classification of adverse events.

Adverse Event	MENOLACTO^®^ (*n* = 42)		Placebo (*n* = 43)	
	Case	Incidence Rate (%)	Case	Incidence Rate (%)
Lymphadenopathy	0	0	1	10.00
Cystitis	1	8.33	0	0
Pneumonia	0	0	1	10.00
Upper respiratory tract infection	1	8.33	0	0
Musculoskeletal pain	0	0	1	10.00
Temporomandibular joint syndrome	1	8.33	0	0
Headache	2	16.67	0	0
Paresthesia	1	8.33	0	0
Depression	0	0	1	10.00
Breast pain	3	25.00	0	0
Vaginal discharge	0	0	1	10.00
Cough	0	0	1	10.00
Osteoclastogenic influenza	1	8.33	0	0
Oropharyngeal pain	0	0	3	30.00
Pharyngitis	1	8.33	1	10.00
Pneumonia	1	8.33	0	0
Total	12	100.00	10	100.00

**Table 6:** Changes in E2 and FSH levels relative to baseline.

Domain		*n*	MENOLACTO®	*n*	Placebo	*p* *
E2 (pg/mL)	Baseline	42	28.10 ± 47.78	43	25.82 ± 43.59	
	Week 12	38	16.51 ± 23.16	38	17.92 ± 23.57	0.8678
FSH (mIU/mL)	Baseline	42	75.29 ± 33.91	43	74.02 ± 30.38	
	Week 12	38	79.64 ± 27.37	38	80.81 ± 37.35	0.7526

Values are presented as the mean ± SD. * Comparison between groups (changes from baseline); two-sample *t*-test.

**Table 7:** Changes in endometrial thickness relative to baseline.

Domain		*n*	MENOLACTO^®^	*n*	Placebo	*p* *
Endometrial thickness (mm)	Baseline	37 ^a^	2.78 ± 1.68	39 ^b^	2.56 ± 1.19	
	Week 12	33	2.42 ± 1.80	34	2.86 ± 1.59	0.2091

Values are presented as the mean ± SD. * Comparison between groups (changes from baseline); two-sample *t*-test. ^a^ MENOLACTO^®^ group: 5 subjects of no uterus were excluded. ^b^ Placebo group: 4 subjects of no uterus were excluded.

## References

[1] Sivamaruthi B.S., Kesika P., Chaiyasut C. (2018). Influence of probiotic supplementation on climacteric symptoms in menopausal women—A mini review. Int. J. Appl. Pharm..

[2] Davis S.R., Lambrinoudaki I., Lumsden M., Mishra G.D., Pal L., Rees M., Santoro N., Simoncini T. (2015). Menopause. Nat. Rev. Dis. Primers.

[3] Lobo R.A. (2019). Menopause and aging. Yen and Jaffe’s Reproductive Endocrinology.

[4] Monteleone P., Mascagni G., Giannini A., Genazzani A.R., Simoncini T. (2018). Symptoms of menopause—Global prevalence, physiology and implications. Nat. Rev. Endocrinol..

[5] Park J., Kim K. (2016). A randomized, double-blind, placebo-controlled trial of Schisandra chinensis for menopausal symptoms. Climacteric.

[6] Johnson A., Roberts L., Elkins G. (2019). Complementary and alternative medicine for menopause. J. Evid. -Based Integr. Med..

[7] Borrelli F., Ernst E. (2010). Alternative and complementary therapies for the menopause. Maturitas.

[8] Stubbs C., Mattingly L., Crawford S.A., Wickersham E.A., Brockhaus J.L., McCarthy L.H. (2017). Do SSRIs and SNRIs reduce the frequency and/or severity of hot flashes in menopausal women. J. Okla. State Med. Assoc..

[9] Yadav M., Volkar J. (2013). Potential role of gabapentin and extended-release gabapentin in the management of menopausal hot flashes. Int. J. Gen. Med..

[10] Cintron D., Lahr B.D., Bailey K.R., Santoro N., Lloyd R., Manson J.E., Neal-Perry G., Pal L., Taylor H.S., Wharton W. (2018). Effects of oral versus transdermal menopausal hormone treatments on self-reported sleep domains and their association with vasomotor symptoms in recently menopausal women enrolled in the Kronos Early Estrogen Prevention Study (KEEPS). Menopause.

[11] Utian W., Yu H., Bobula J., Mirkin S., Olivier S., Pickar J.H. (2009). Bazedoxifene/conjugated estrogens and quality of life in postmenopausal women. Maturitas.

[12] Archer D.F., Kimble T.D., Lin F.Y., Battucci S., Sniukiene V., Liu J.H. (2018). A randomized, multicenter, double-blind, study to evaluate the safety and efficacy of estradiol vaginal cream 0.003% in postmenopausal women with vaginal dryness as the most bothersome symptom. J. Women’s Health.

[13] Eriksen P.S., Rasmussen H. (1992). Low-dose 17β-estradiol vaginal tablets in the treatment of atrophic vaginitis: A double-blind placebo controlled study. Eur. J. Obstet. Gynecol. Reprod. Biol..

[14] Lambert M.N.T., Thorup A.C., Hansen E.S.S., Jeppesen P.B. (2017). Combined Red Clover isoflavones and probiotics potently reduce menopausal vasomotor symptoms. PLoS ONE.

[15] Imhof M., Gocan A., Imhof M., Schmidt M. (2018). Soy germ extract alleviates menopausal hot flushes: Placebo-controlled double-blind trial. Eur. J. Clin. Nutr..

[16] Mohammad-Alizadeh-Charandabi S., Shahnazi M., Nahaee J., Bayatipayan S. (2013). Efficacy of black cohosh (Cimicifuga racemosa L.) in treating early symptoms of menopause: A randomized clinical trial. Chin. Med..

[17] Hu L., Yue Y., Zuo P.-P., Jin Z.-Y., Feng F., You H., Li M.-L., Ge Q.-S. (2006). Evaluation of neuroprotective effects of long-term low dose hormone replacement therapy on postmenopausal women brain hippocampus using magnetic resonance scanner. Chin. Med. Sci. J..

[18] Rasgon N.L., Geist C.L., Kenna H.A., Wroolie T.E., Williams K.E., Silverman D.H. (2014). Prospective randomized trial to assess effects of continuing hormone therapy on cerebral function in postmenopausal women at risk for dementia. PLoS ONE.

[19] MacDonald A., Murphy E., Capell H., Bankowska U., Ralston S. (1994). Effects of hormone replacement therapy in rheumatoid arthritis: A double blind placebo-controlled study. Ann. Rheum. Dis..

[20] Gambacciani M., Levancini M. (2014). Hormone replacement therapy and the prevention of postmenopausal osteoporosis. Prz. Menopauzalny.

[21] Vinogradova Y., Coupland C., Hippisley-Cox J. (2019). Use of hormone replacement therapy and risk of venous thromboembolism: Nested case-control studies using the QResearch and CPRD databases. BMJ.

[22] Windler E., Stute P., Ortmann O., Mueck A.O. (2015). Is postmenopausal hormone replacement therapy suitable after a cardio-or cerebrovascular event?. Arch. Gynecol. Obs..

[23] Hou N., Hong S., Wang W., Olopade O.I., Dignam J.J., Huo D. (2013). Hormone replacement therapy and breast cancer: Heterogeneous risks by race, weight, and breast density. J. Natl. Cancer Inst..

[24] Peng C.-C., Liu C.-Y., Kuo N.-R., Tung T.-H. (2019). Effects of Phytoestrogen Supplement on Quality of Life of Postmenopausal Women: A Systematic Review and Meta-Analysis of Randomized Controlled Trials. Evid. Based Complement. Altern. Med..

[25] Seidl M.M., Stewart D.E. (1998). Alternative treatments for menopausal symptoms. Systematic review of scientific and lay literature. Can. Fam. Physician.

[26] Mohajeri M.H., Brummer R.J., Rastall R.A., Weersma R.K., Harmsen H.J., Faas M., Eggersdorfer M. (2018). The role of the microbiome for human health: From basic science to clinical applications. Eur. J. Nutr..

[27] Durack J., Lynch S.V. (2019). The gut microbiome: Relationships with disease and opportunities for therapy. J. Exp. Med..

[28] Martin C.R., Osadchiy V., Kalani A., Mayer E.A. (2018). The brain-gut-microbiome axis. Cell. Mol. Gastroenterol. Hepatol..

[29] Takada M., Nishida K., Gondo Y., Kikuchi-Hayakawa H., Ishikawa H., Suda K., Kawai M., Hoshi R., Kuwano Y., Miyazaki K. (2017). Beneficial effects of Lactobacillus casei strain Shirota on academic stress-induced sleep disturbance in healthy adults: A double-blind, randomised, placebo-controlled trial. Benef. Microbes.

[30] Karakula-Juchnowicz H., Rog J., Juchnowicz D., Łoniewski I., Skonieczna-Żydecka K., Krukow P., Futyma-Jedrzejewska M., Kaczmarczyk M. (2019). The study evaluating the effect of probiotic supplementation on the mental status, inflammation, and intestinal barrier in major depressive disorder patients using gluten-free or gluten-containing diet (SANGUT study): A 12-week, randomized, double-blind, and placebo-controlled clinical study protocol. Nutr. J..

[31] Zhang J., Lu Y., Wang Y., Ren X., Han J. (2018). The impact of the intestinal microbiome on bone health. Intractable Rare Dis. Res..

[32] Vieira A.T., Castelo P.M., Ribeiro D.A., Ferreira C.M. (2017). Influence of oral and gut microbiota in the health of menopausal women. Front. Microbiol..

[33] Flores R., Shi J., Fuhrman B., Xu X., Veenstra T.D., Gail M.H., Gajer P., Ravel J., Goedert J.J. (2012). Fecal microbial determinants of fecal and systemic estrogens and estrogen metabolites: A cross-sectional study. J. Transl. Med..

[34] Lim E., Kim J., Jung S., Song E., Lee S., Shin H., Nam Y., Kim Y. (2019). Attenuating Effects of Lactobacillus acidophilus YT1 on Menopausal Symptoms in Ovariectomized Rats. J. Korean Soc. Food Sci. Nutr..

[35] Lee M., Yun J.M., Park S.J., Kim D.K., Kim O.K. (2019). Effect of Lactobacillus acidophilus YT1 on Serum Lipid and Bone Formation in Ovariectomized Rats. J. Korean Soc. Food Sci. Nutr..

[36] Alder E. (1998). The Blatt-Kupperman menopausal index: A critique. Maturitas.

[37] Lee J., Kim K.W., Kim H.-K., Chae S.-W., Jung J.-C., Kwon S.H., Rheu C.H. (2010). The effect of Rexflavone (Sophorae fructus extract) on menopausal symptoms in postmenopausal women: A randomized double-blind placebo controlled clinical trial. Arch. Pharmacal Res..

[38] Chang A., Kwak B.Y., Yi K., Kim J.S. (2012). The effect of herbal extract (EstroG-100) on pre-, peri-and post-menopausal women: A randomized double-blind, placebo-controlled study. Phytother. Res..

[39] Hilditch J.R., Lewis J., Peter A., van Maris B., Ross A., Franssen E., Guyatt G.H., Norton P.G., Dunn E. (1996). A menopause-specific quality of life questionnaire: Development and psychometric properties. Maturitas.

[40] Warren M.P., Shu A.R., Dominguez J.E. (2015). Menopause and hormone replacement. Endotext [Internet].

[41] Humphries K.H., Gill S. (2003). Risks and benefits of hormone replacement therapy: The evidence speaks. CMAJ.

[42] Tanmahasamut P., Vichinsartvichai P., Rattanachaiyanont M., Techatraisak K., Dangrat C., Sardod P. (2015). Cimicifuga racemosa extract for relieving menopausal symptoms: A randomized controlled trial. Climacteric.

[43] Husain D., Khanna K., Puri S., Haghighizadeh M. (2015). Supplementation of soy isoflavones improved sex hormones, blood pressure, and postmenopausal symptoms. J. Am. Coll. Nutr..

[44] Chen M., Lin C., Liu C. (2015). Efficacy of phytoestrogens for menopausal symptoms: A meta-analysis and systematic review. Climacteric.

[45] Lethaby A., Marjoribanks J., Kronenberg F., Roberts H., Eden J., Brown J. (2007). Phytoestrogens for vasomotor menopausal symptoms. Cochrane Database Syst. Rev..

[46] Jeong S.-Y., Kang S., Hua C.S., Ting Z., Park S. (2017). Synbiotic effects of β-glucans from cauliflower mushroom and Lactobacillus fermentum on metabolic changes and gut microbiome in estrogen-deficient rats. Genes Nutr..

[47] Zhang Z., Chen Y., Xiang L., Wang Z., Xiao G.G., Hu J. (2017). Effect of curcumin on the diversity of gut microbiota in ovariectomized rats. Nutrients.

[48] Sanders M.E., Merenstein D.J., Reid G., Gibson G.R., Rastall R.A. (2019). Probiotics and prebiotics in intestinal health and disease: From biology to the clinic. Nat. Rev. Gastroenterol. Hepatol..

[49] Pickard J.M., Zeng M.Y., Caruso R., Núñez G. (2017). Gut microbiota: Role in pathogen colonization, immune responses, and inflammatory disease. Immunol. Rev..

[50] Choi S., Hwang Y.-J., Shin M.-J., Yi H. (2017). Difference in the gut microbiome between Ovariectomy-induced obesity and diet-induced obesity. J. Microbiol. Biotechnol..

[51] Santos-Marcos J.A., Rangel-Zuñiga O.A., Jimenez-Lucena R., Quintana-Navarro G.M., Garcia-Carpintero S., Malagon M.M., Landa B.B., Tena-Sempere M., Perez-Martinez P., Lopez-Miranda J. (2018). Influence of gender and menopausal status on gut microbiota. Maturitas.

[52] Li J.-Y., Chassaing B., Tyagi A.M., Vaccaro C., Luo T., Adams J., Darby T.M., Weitzmann M.N., Mulle J.G., Gewirtz A.T. (2016). Sex steroid deficiency–associated bone loss is microbiota dependent and prevented by probiotics. J. Clin. Investig..

[53] Chen K.L.A., Liu X., Zhao Y.C., Hieronymi K., Rossi G., Auvil L.S., Welge M., Bushell C., Smith R.L., Carlson K.E. (2018). Long-Term Administration of conjugated estrogen and Bazedoxifene decreased murine fecal β-glucuronidase activity without impacting overall microbiome community. Sci. Rep..

[54] Sovijit W.N., Sovijit W.E., Pu S., Usuda K., Inoue R., Watanabe G., Yamaguchi H., Nagaoka K. (2019). Ovarian progesterone suppresses depression and anxiety-like behaviors by increasing the Lactobacillus population of gut microbiota in ovariectomized mice. Neurosci. Res..

[55] Zhang B., Wang J., Wei Q., Liu Y., Zhang H., Chen X., Xu K. (2020). Epigallocatechin-3-O-gallate modulates the diversity of gut microbiota in ovariectomized rats. Food Sci. Nutr..

[56] McCabe L.R., Irwin R., Schaefer L., Britton R.A. (2013). Probiotic use decreases intestinal inflammation and increases bone density in healthy male but not female mice. J. Cell. Physiol..

[57] Liang S., Wu X., Hu X., Wang T., Jin F. (2018). Recognizing depression from the microbiota–gut–brain axis. Int. J. Mol. Sci..

[58] Petricevic L., Unger F.M., Viernstein H., Kiss H. (2008). Randomized, double-blind, placebo-controlled study of oral lactobacilli to improve the vaginal flora of postmenopausal women. Eur. J. Obstet. Gynecol. Reprod. Biol..

[59] Jaisamrarn U., Triratanachat S., Chaikittisilpa S., Grob P., Prasauskas V., Taechakraichana N. (2013). Ultra-low-dose estriol and lactobacilli in the local treatment of postmenopausal vaginal atrophy. Climacteric.

[60] Jansson P.-A., Curiac D., Ahrén I.L., Hansson F., Niskanen T.M., Sjögren K., Ohlsson C. (2019). Probiotic treatment using a mix of three Lactobacillus strains for lumbar spine bone loss in postmenopausal women: A randomised, double-blind, placebo-controlled, multicentre trial. Lancet Rheumatol..

[61] Marotta A., Sarno E., Del Casale A., Pane M., Mogna L., Amoruso A., Felis G.E., Fiorio M. (2019). Effects of probiotics on cognitive reactivity, mood and sleep quality. Front. Psychiatry.

[62] Wallace C.J., Foster J.A., Soares C.N., Milev R.V. (2020). The effects of probiotics on symptoms of depression: Protocol for a double-blind randomized placebo-controlled trial. Neuropsychobiology.

[63] Roman P., Carrillo-Trabalon F., Sanchez-Labraca N., Cañadas F., Estevez A., Cardona D. (2018). Are probiotic treatments useful on fibromyalgia syndrome or chronic fatigue syndrome patients? A systematic review. Benef. Microbes.

[64] Dai Y.-J., Wang H.-Y., Wang X.-J., Kaye A.D., Sun Y.-H. (2017). Potential beneficial effects of probiotics on human migraine headache: A literature review. Pain Physician.

[65] Lu M., Zhou Y., Wang B., Hu Z., Du Y., Liu S., Lin X., Cui Y., Jin H. (2018). Impact of multidisciplinary collaborative pharmaceutical care on knowledge, adherence, and efficacy of hormone therapy in climacteric women. Patient Prefer. Adherence.

